# P-736. Evaluation of virologic rebounds in PLWH after receiving MVA-BN vaccination for mpox

**DOI:** 10.1093/ofid/ofaf695.947

**Published:** 2026-01-11

**Authors:** Pierluigi Francesco Salvo, Gianmaria Baldin, Francesca Lombardi, Valeria Campolattano, Valentina Iannone, Andrea Carbone, giulia lenzi, Rebecca Jo Steiner, Carlo Torti, Simona Di Giambenedetto

**Affiliations:** Università Cattolica del Sacro Cuore, Roma, Lazio, Italy; Fondazione Policlinico A. Gemelli IRCCS, Roma, Lazio, Italy; Fondazione Policlinico A. Gemelli IRCCS, Roma, Lazio, Italy; Università Cattolica del Sacro Cuore, Roma, Lazio, Italy; Università Cattolica del Sacro Cuore, Roma, Lazio, Italy; Università Cattolica del Sacro Cuore, Roma, Lazio, Italy; Università Cattolica del Sacro Cuore, Roma, Lazio, Italy; Università Cattolica del Sacro Cuore, Roma, Lazio, Italy; Università Cattolica del Sacro Cuore, Roma, Lazio, Italy; Università Cattolica del Sacro Cuore, Roma, Lazio, Italy

## Abstract

**Background:**

The Mpox vaccination with modified vaccinia Ankara-Bavarian Nordic (MVA-BN) has emerged as a critical intervention for preventing disease spread. However, its potential impact on viral load dynamics in people living with HIV (PLWH) remains unclear. This study aims to evaluate whether MVA-BN is associated with the occurrence of virological rebound [(VR), viral blips (VB) or virologic failure (VF)] in PLWH within 6 months following vaccination

**Table 1:**
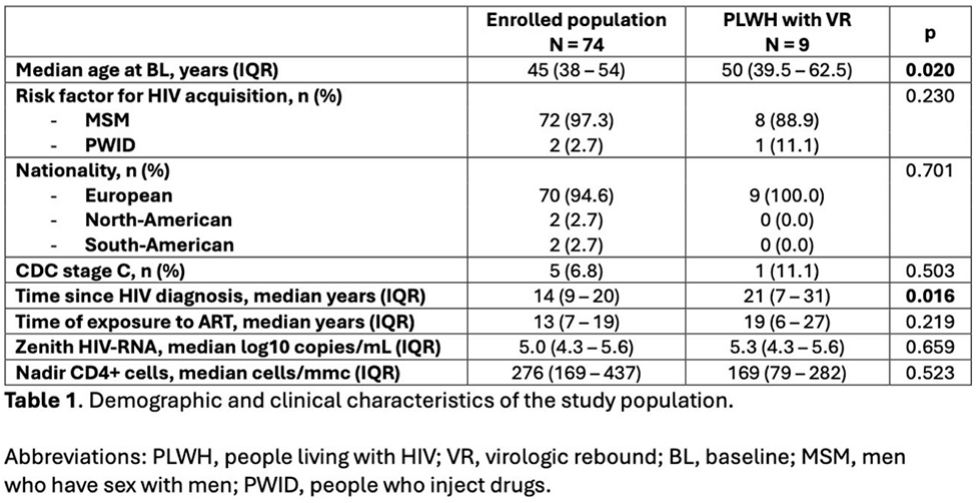
Demographic and clinical characteristics of the study population

**Methods:**

We enrolled all PLWH who were vaccinated with MVA-BN and had maintained stable virological suppression (target non detectable, TND) for at least 12 months prior to vaccination. Participants were required to be on the same ART for at least 6 months, have at least 200 CD4+ cells/mmc at the time of vaccination, and have at least 1 determination of HIV-RNA within 6 months after completing the vaccination cycle. VB were defined as a single determination of HIV-RNA > 50 cp/mL followed by a determination < 50 cp/mL; VF was defined as a single determination > 1000 cp/mL or at least 2 consecutive measurements > 50 cp/mL. Time to VR was estimated using Kaplan-Meier analysis, and a Cox regression analysis was conducted to evaluate the factors associated with the occurrence of VR

**Results:**

A total of 74 PLWH, all male, were enrolled. The characteristics of the population are summarized in Table1. We observed 8 VB (incidence rate 1.93 per 100 PMFU, 95%CI 0.59 – 3.27), with all events exceeding 100 copies/mL, and 1 VF (incidence rate 0.24 per 100 PMFU, 95% CI 0.00 – 0.71) with 7410 copies/mL. Previous low-level viremia (LLV, defined as at least 2 consecutive measurements > 50 cp/mL and < 200 cp/mL) occurring between 3 and 1 year before vaccination, independently predicted VR after vaccination in a multivariate regression analysis (aHR 13.8; 95%CI 2.2 – 88.0; p = 0.005). For all events, adherence issues were excluded, and all participants returned to a TND status at the subsequent determination without any modifications to their ART regimen

**Conclusion:**

Although rare, the occurrence of VR following MVA-BN in PLWH suggests the need for careful monitoring of HIV-RNA levels after vaccination, especially in individuals with a history of viral load fluctuations. Further research is needed to elucidate the underlying mechanisms and assess the clinical and epidemiological implications

**Disclosures:**

All Authors: No reported disclosures

